# Compositions, Antimicrobial, and Antioxidant Activities of the Essential Oil Extracted From Garden Cress Seeds Growing in Three Districts of South Wollo, Ethiopia

**DOI:** 10.1002/fsn3.71119

**Published:** 2025-10-22

**Authors:** Melese Damtew Asfaw, Mequanint Gebeyehu Awoke, Adamu Tizazu Yadeta

**Affiliations:** ^1^ College of Natural and Computational Science Mekdela Amba University Tulu Awuliya Ethiopia

**Keywords:** antimicrobial activity, antioxidant activity, chemical composition, essential oil, *Lepidium sativum*

## Abstract

This study explores the chemical composition, antimicrobial, and antioxidant properties of essential oils derived from *Lepidium sativum* seeds, collected from Legambo, Jama, and Tenta districts. Notably, the oils were predominantly composed of benzyl nitrile, alongside sulfur‐containing compounds like trisulfide bis(phenylmethyl) and disulfide bis(phenylmethyl). The essential oil yield varied between 2.3% and 3.8%, with the Tenta sample yielding the highest amount. The oils demonstrated significant antimicrobial activity, with inhibition zones ranging from 27.2 to 36.8 mm and minimum inhibitory concentration (MIC) values of 1.26–3.00 mg/mL for bacterial strains and 2.01–2.62 mg/mL for fungal strains. Remarkably, the strongest antimicrobial effect was against *Staphylococcus aureus* (MIC = 1.26 mg/mL) and *Candida albicans* (MIC = 2.01 mg/mL). Additionally, the essential oils exhibited robust antioxidant potential, with the oil from the Tenta district showing the highest activity, as indicated by an IC_50_ value of 11.31 ± 0.01 μg/mL in the DPPH radical scavenging assay. Significant variations in essential oil composition and bioactivity were observed across districts, suggesting that environmental factors such as soil type, climate, and altitude play critical roles in shaping these differences. This study not only highlights the therapeutic potential of *L. sativum* essential oils in antimicrobial and antioxidant applications but also underscores the importance of investigating district‐specific variations and the commercial viability of these bioactive components. The findings provide a novel contribution to the understanding of *L. sativum* 's bioactive properties and their implications for health and industry.

## Graphical abstract

This study analyzes the chemical composition and evaluates the antimicrobial and antioxidant activities of essential oils extracted from *Lepidium sativum* (garden cress) seeds collected from three districts in South Wollo, Ethiopia. The oils were rich in benzyl nitrile and sulfur‐containing compounds, demonstrating significant bioactive potential. The findings suggest the essential oils possess promising natural antimicrobial and antioxidant properties.

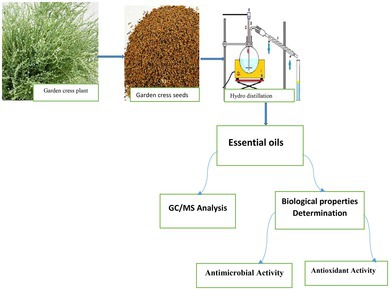

## 1 Introduction

Herbs and spices have been used since ancient times to enhance the flavor and organoleptic properties of various foods. Additionally, herbal medicines hold significant potential in the growing nutrition industry, as these substances are often considered both foods and medicines. They are widely utilized for preventive and curative treatments around the world (Lawrence 2007). Today, the concept of “functional foods,” which combines nutritional and medicinal benefits, is especially popular. Many natural compounds derived from plants have shown biological activities. Among these natural substances, essential oils from aromatic and medicinal plants are of particular interest as potential natural agents for food preservation (Nikšić et al. 2012).

Essential oils are volatile organic compounds present in various plant tissues, including fruits, leaves, flowers, bark, stems, seeds, wood, and roots (Ben Ghnaya et al. 2013). These oils are complex chemical mixtures made up of several different types of molecules. Most of the volatile components in essential oils are terpenoid derivatives (Emami et al. 2011). A wide range of biological activities has been reported for essential oils, including antibacterial, antiviral, antifungal, anticancer, anticonvulsant, spasmolytic, expectorant, immunomodulatory, and antidiabetic properties (Ismaili et al. 2001).

In addition to the properties mentioned above, many essential oils have been shown to possess antioxidant activity. This is crucial for disease prevention, as antioxidants help inhibit and delay the oxidation of biomolecules by preventing the initiation or propagation of chain oxidation reactions (Quariachi et al. 2011). Given the harmful effects associated with synthetic antioxidants, such as toxicity and carcinogenicity, there has been a significant increase in interest in discovering natural antioxidants (Losso et al. 2007).

The increasing interest in natural bioactive compounds has spurred extensive research aimed at replacing synthetic chemical agents across various industries. This shift is driven by the recognition that natural products tend to be less harmful to human health compared to their synthetic counterparts (Gao et al. 2011). Furthermore, natural compounds are often biodegradable, making them more environmentally friendly and sustainable. Their low toxicity in mammals further enhances their appeal, as they present fewer health risks in both short‐ and long‐term exposure (Figueiredo et al. 2008). As a result, many industries are exploring the potential of natural bioactive substances as safer, more effective alternatives to synthetic chemicals.

Plant‐based drugs, as alternative antibiotics, are being increasingly investigated as potential replacements for traditional antibiotics. This growing interest has led to substantial research aimed at evaluating the antimicrobial effects of essential oils and extracts. These natural substances have shown promising potential in inhibiting the growth of a wide range of harmful microorganisms, offering a viable alternative to conventional antimicrobial agents (Crepaldi et al. 2022).

*Lepidium sativum* , commonly known as garden cress, belongs to the Cruciferae (Brassicaceae) family (Agarwal and Sharma 2013; Singh et al. 2015). In Ethiopia, it is locally called “Feto” and is believed to have originated in the country (Nadkarni 1954). All parts of the garden cress plant, including seeds, leaves, and roots, hold significant economic value (Agarwal and Sharma 2013). However, it is primarily cultivated for its seeds (Kapseu and Parmentier 1997). Garden cress seeds are known for a variety of medicinal properties, including being galactagogue, bitter, thermogenic, depurative, rubefacient, aphrodisiac, ophthalmic, antiscorbutic, antihistaminic, diuretic, and tonic (Mohite et al. 2012). The seeds are used to treat a wide range of ailments, including asthma, coughs with expectoration, diarrhea, dysentery, sprains, leprosy, skin diseases, splenomegaly, dyspepsia, lumbago, leucorrhea, scurvy, and seminal weakness (Mali et al. 2007).

In many parts of Ethiopia, a traditional dish called “Feto Fitfit” is prepared using the seeds of *L. sativum* . Despite the widespread use of *L. sativum* in local practices and the plant's diverse chemical composition, limited research has been conducted within the country. In folk medicine, *L. sativum* is commonly used to treat inflammatory conditions such as diabetes mellitus, arthritis, and hepatitis (Sarkar et al. 2014). Several studies have highlighted that extracts of *L. sativum* exhibit a range of beneficial effects, including antioxidant, antidiarrheal, antispasmodic, antimicrobial, anti‐inflammatory, and hepatoprotective properties, particularly in combating oxidative damage (Raish et al. 2016).

In Ethiopia, while *L. sativum* is commonly used in various food preparations, as a spice, and for its traditional medicinal properties, no research has been conducted on the chemical composition, antimicrobial, or antioxidant activities of the essential oil extracted from *L. sativum* seeds. Therefore, the present study aims to investigate the chemical composition of the essential oil derived from *L. sativum* seeds, evaluate its antimicrobial potential against a range of microorganisms (including two Gram‐negative bacteria, two Gram‐positive bacteria, and one dermatophytic fungus), and assess its antioxidant activity.

## 2 Experimental

### 2.1 Apparatus and Equipment

The analytical instruments employed in this study, along with their corresponding models and manufacturers, are summarized in Table 1. These instruments were selected to ensure accurate and reliable measurement across different analyses. Proper calibration and maintenance procedures were followed according to the manufacturer's guidelines to guarantee precision and reproducibility of the results.

**TABLE 1:** List of analytical instruments used in the study, including their model numbers and manufacturers.

Instrument	Model	Manufacturer
GC/MS	Agilent 5975N	Agilent Technologies, California, USA
GC	Shimadzu GC‐2010	Shimadzu Corporation, Kyoto, Japan
UV–VIS spectrophotometer	U‐1789	Secommsan, France
Polarimeter	D7	Bellingham & Stanley, UK
Pycnometer	290	Erichsen, Germany
Abbe refractometer	AR2008	A. Kruss, Germany
Electronic balance	SN 8732351170	Ohaus, USA/China
Petri plates	—	Biochek Lab., Greece
Hot air oven	IM‐30m	Irmeco, Germany

### 2.2 Chemicals

The ascorbic acid (99.9% purity) used in this study was sourced from Naarden International U.K. Limited, England. Polyoxyethylene (20) sorbitan monooleate (Tween 80), dimethyl sulfoxide (DMSO), barium chloride (BaCl_2_), and methanol were procured from Alpha Chemika, Mumbai, India. Sodium chloride was supplied by Cook's Company, Egypt, and 2,2‐diphenyl‐1‐picrylhydrazyl (DPPH) and anhydrous sodium sulfate were obtained from Sigma‐Aldrich, Germany. The bacterial strains *Staphylococcus aureus* , *Bacillus subtilis* , *Escherichia coli* , *Pasteurella multocida* , and the fungal strain *Candida albicans* were kindly provided by Bless Agri Food Laboratory Services PLC, Addis Ababa, Ethiopia. Microbiological media, including Nutrient Agar, Mueller Hinton Agar, Mueller Hinton Broth for bacterial growth, as well as Potato Dextrose Agar and Potato Dextrose Broth for fungal growth, were purchased from Oxoid Ltd., Cairo, Egypt.

### 2.3 Raw Material Preparation

**FIGURE 1:**
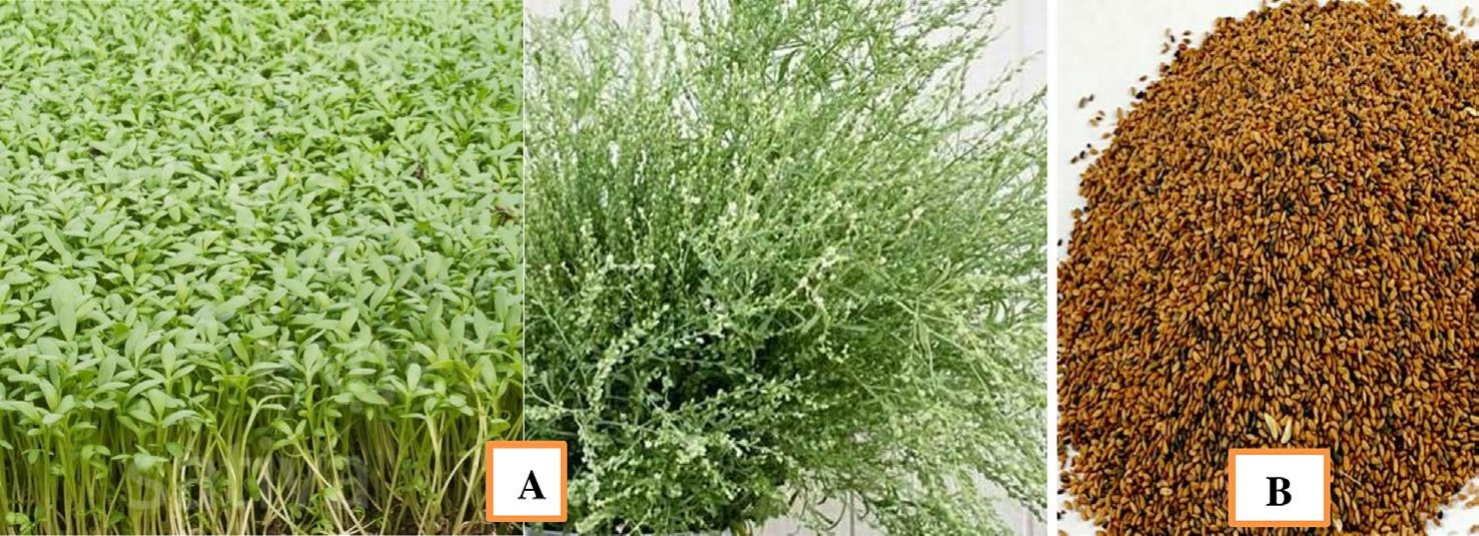
*Lepidium sativum* (garden cress) plant and seeds. (a) Whole garden cress plant showing vegetative growth, and (b) cleaned seeds collected from Jama, Legambo, and Tenta Woreda, South Wollo, Amhara Regional State, Ethiopia (geographical coordinates provided in the text). Voucher specimen No. 0007 is deposited at the National Herbarium, Addis Ababa University.

The *Lepidium sativum* seeds were collected in January 2024 from three locations in South Wollo, Amhara Regional State, Ethiopia: Jama (10°38′2″ N, 39°19′53″ E), Legambo (10°53′56″ N, 39°3′58″ E), and Tenta Woreda (11°14′22″ N, 39°14′38″ E). The seeds were carefully cleaned to remove dust, dirt, foreign materials, and broken seeds to ensure sample quality. Plant material identification was authenticated by taxonomist Melaku Gizachew, and a voucher specimen (Voucher No. 0007) was deposited at the National Herbarium, Department of Biology, Addis Ababa University, Addis Ababa, Ethiopia (Figure 1).

### 2.4 Extraction of Essential Oil

Dried and pulverized seeds of *L. sativum* (100 g) were submitted to hydrodistillation (HD) for 3 h with 500 mL distilled water using a Clevenger‐type apparatus according to the European Pharmacopeia. The extracted oil was collected and dried over anhydrous sodium sulfate, then stored in sealed glass vials in a refrigerator at 4°C before analysis.

The yield of the essential oil was calculated based on the dry weight of the plant material. The percent yield of the essential oil was determined using the following formula:

$$\text{Essential oil yield}\;\left(\%\right)=\frac{\;\text{Amount of essential oil extracted}\;\left(\mathrm{mL}\right)\;}{\text{Mass of seed used}\;\left(g\right)}\times 100$$

All measurements were carried out in triplicate, and the mean values were determined.

### 2.5 Analysis of the Essential Oil

#### 2.5.1 Physical Analyses

The refractive index of *L. sativum* seeds essential oil at 25°C was measured using an Abe refractometer at a wavelength of 632.8 nm. The refractive index was calculated using the following formula (Guenther 1961):

$$N=\tan\;\left(\mathrm{\theta b}\right)$$

where *N* is the refractive index and (*θb*) is the angle of rotation; (*θb*) = rotation with sample—initial rotation.

The optical rotation of the essential oil was measured using a polarimeter with a 2 dm sample tube. The angle of rotation was recorded at 25°C using the D‐line of polarized sodium light, which has a wavelength of 589 nm.

The specific gravity of the essential oil was determined using a Pycnometer tube of known weight (*W*_1_). The procedure involved first filling the Pycnometer with essential oil and measuring the weight (*W*_2_), followed by filling the Pycnometer with water and measuring the weight (*W*_3_). The specific gravity of the oil was then calculated using the following formula (Guenther 1961):

$$\text{Specific gravity}=\frac{w_{2}-w_{1}}{w_{3}-w_{1}}$$

where

*W*_2_ = mass of the pycnometer + essential oil (in grams).

*W*_1_ = mass of the empty pycnometer (in grams).

*W*_3_ = mass of the pycnometer + water (in grams).

#### 2.5.2 Chromatographic Analysis

Qualitative and quantitative analyses of the essential oils were conducted using a gas chromatography–mass spectrometry (GC–MS) system (Agilent Technologies series 6890N/5975B, United States) with an electron energy of 70 eV. The system was equipped with a split‐splitless injector (200°C) and a flame ionization detector (FID) (250°C). Helium (1 mL/min) was used as the carrier gas. Capillary columns (HP 5MS 30 m × 0.25 mm; film thickness 0.25 μm, Agilent Technologies) were employed. The temperature program was set from 50°C to 280°C at a rate of 10°C/min until 130°C, and from 130°C to 280°C at a rate of 12°C/min, with a split ratio of 1:10. Coelution and mass spectrometry (MS) analysis involved the identification of individual compounds by comparing their relative retention indices (RI) with those of reference samples. For components, mostly sesquiterpenes and aliphatic compounds, for which reference substances were unavailable, identification was based on matching retention times and mass spectra with those obtained from authentic samples and/or the National Institute of Standards and Technology (NIST) and Wiley library spectra, as well as literature data (Adams 2001).

### 2.6 Determination of the Antimicrobial Activity of the Essential Oil

The antimicrobial activities of all samples were tested against five test microorganisms: two Gram‐positive bacteria, *Staphylococcus aureus* (ATCC 31488) and *Bacillus subtilis* (ATCC 6633); two Gram‐negative bacteria, *Escherichia coli* (ATCC 25922) and *Pasteurella multocida* (ATCC 9027); and one dermatophytic fungus, *Candida albicans* (FMC17).

### 2.7 Preparation of Culture Media

Potato Dextrose Agar (PDA) and Potato Dextrose Broth (PDB) were used for antifungal bioassays, while Muller‐Hinton Agar (MHA) and Muller‐Hinton Broth (MHB) were used for antibacterial bioassays. Nutrient Agar (NA) was used for bacterial culture maintenance. The media were prepared as follows: MHB was dissolved in 1 L of water, and 28 g of NA was dissolved in 1 L of water. The media were thoroughly mixed by stirring until completely dissolved, then sterilized by autoclaving at 121°C and 103,421.4 Pa for 15 min.

For the preparation of Potato Dextrose Broth, a potato infusion was made by boiling 200 g of sliced, unpeeled potatoes in 1 L of distilled water for 30 min. The mixture was then filtered through double‐layer cheesecloth to collect the effluent. The potato infusion was combined with 20 g of dextrose and autoclaved at 121°C and 103,421.4 Pa for 15 min. The final pH was adjusted to 5.6, and the medium was stored in tightly closed bottles.

After autoclaving, the agar media were allowed to cool to 45°C, then dispensed into flat‐bottomed Petri dishes to solidify at room temperature (23°C ± 2°C). All media were stored in screw‐capped bottles in a refrigerator until needed (Souza et al. 2005; National Committee for Clinical Laboratory Standards 2002).

### 2.8 Retrieval of the Test Pathogen and Preparation of Inoculation

The stock culture of the test fungal pathogen, maintained at −20°C, was retrieved by subculturing onto Potato Dextrose Agar (PDA). A well‐isolated, pure colony of the same morphological type was selected from the agar plate. Using a sterile loop, the top mycelia of the colony were picked and transferred into a test tube containing 10 mL of Potato Dextrose Broth (PDB). The culture tube was incubated for 5 days at room temperature (23°C ± 2°C) to obtain fresh fungal cultures.

The turbidity of the fungal suspensions was adjusted to the required range using the McFarland standard. To prepare the 0.5 McFarland turbidity standard, 0.05 mL of 1% BaCl_2_ dehydrate was mixed with 9.95 mL of 1% H_2_SO_4_. Fungal suspensions were prepared in sterile saline (0.85% NaCl wt/vol) by dissolving 0.85 g of NaCl in 100 mL of distilled water and autoclaving for 15 min at 121°C and 103,421.4 Pa. The turbidity of the fungal suspensions was then adjusted to 0.5 McFarland, equivalent to 1 × 10^6^ Colony Forming Units (CFU)/mL.

For bacterial test pathogens, stock cultures of the three bacterial species, maintained at −20°C, were retrieved by subculturing onto Nutrient Agar (NA) plates. Three well‐isolated pure colonies of the same morphological type were selected from the NA plates and aseptically transferred into test tubes containing 10 mL of Muller‐Hinton Broth (MHB) using a sterile loop. The culture tubes were incubated at 37°C for 24 h to obtain fresh bacterial cultures.

The turbidity of the bacterial suspensions for the bioassays was adjusted to 0.5 McFarland standards, equivalent to 1.5 × 10^8^ CFU/mL (National Committee for Clinical Laboratory Standards 2002).

### 2.9 Preparation and Sterilization of Paper Discs

Paper discs for use in the bioassays were prepared as follows: using a paper punch, 6 mm diameter discs were cut from Whatman filter paper (No. 1). The paper discs were placed in a clean, dry, capped universal bottle and autoclaved for 15 min at 121°C and 103,421.4 Pa. After autoclaving, the paper discs were stored in a sterile condition until needed for use.

### 2.10 Assessment of the Antifungal Activity of the Essential Oil

The antifungal activity of *L. sativum* seed essential oil against the tested fungal pathogens was evaluated using the disc diffusion method, also known as the Kirby‐Bauer antimicrobial susceptibility test, as described by Souza et al. (2005). A 7‐day‐old culture grown on Potato Dextrose Agar (PDA) in the dark to promote sporulation was used to prepare a spore suspension. The suspension was adjusted to an optical density equivalent to 0.5 McFarland standards. To enhance the dispersion of spores, one drop of Tween 20 per mL (approximately 0.01–0.02 mL or 0.5%–1%) was added, as recommended by NCCLS (National Committee for Clinical Laboratory Standards 2002).

Approximately 200 μL of the standardized spore suspension was evenly spread on Petri plates containing PDA medium using a sterile L‐shaped glass rod. Sterile No. 1 Whatman filter paper discs were impregnated with 10 μL of undiluted essential oil, which was then aseptically placed at the center of the inoculated plates. Dimethyl sulfoxide (DMSO) was used as a negative control, while Apron Star (Thiamethoxam 200 g/kg, Mefenoxam 200 g/kg, and Difenoconazole 20 g/kg), a broad‐spectrum seed treatment fungicide, was used as a positive control according to the manufacturer's instructions.

The Petri dishes were kept in a refrigerator at 4°C for 2 h to allow the essential oils to diffuse into the agar medium. The plates were then incubated at room temperature (23°C ± 2°C), and fungal growth was monitored from the 3rd to the 14th day. The diameters of the inhibition zones were measured on the 7th day, as preliminary studies (Souza et al. 2005) showed that fungal growth was still occurring on the 3rd day, but oil activity was at its peak between the 5th and 7th days. On the 14th day, however, the oil activity had decreased, and fungal overgrowth was observed. The tests were conducted in triplicate.

### 2.11 Assessment of the Antibacterial Activity of the Essential Oil

The antibacterial activity of *L. sativum* seed essential oil against the test bacterial pathogens was similarly assessed using the disc diffusion method. A bacterial suspension prepared from an overnight culture was adjusted to an optical density equivalent to 0.5 McFarland standards. About 200 μL of this bacterial suspension was uniformly spread on Petri dishes containing Muller‐Hinton Agar (MHA) using a sterile L‐shaped glass rod. Sterile No. 1 Whatman filter paper discs were each impregnated with 10 μL of undiluted *L. sativum* seed essential oil. The discs were then aseptically placed at the center of the inoculated culture plates. Dimethyl sulfoxide (DMSO) was used as a negative control, while Enrich BM (immunomodulator 2‐Bromo‐2‐Nitropropane‐1,3‐diol), a broad‐spectrum bactericide used in the control of bacterial diseases such as halo blight, bacterial wilt, and bacterial spot, was used as a positive control.

The plates were refrigerated at 4°C for 2 h to allow the essential oil to diffuse into the agar medium. They were then incubated upside down at 37°C for 24 h. The inhibition zones were measured after 24 h. All tests were conducted in triplicate.

For both the antifungal and antibacterial activity tests, the sensitivity of individual microorganisms to the essential oil was classified based on the diameter of the inhibition zone (in millimeters) as follows: not sensitive (−) for total zone diameters less than 8 mm, sensitive (+) for diameters between 8 and 14 mm, very sensitive (++) for zone diameters between 15 and 19 mm, and extremely sensitive (+++) for zone diameters equal to or larger than 20 mm (Celikel and Kavas 2008; Babu et al. 2011).

All tests were conducted in a biological safety cabinet and in accordance with the protocols of the Clinical and Laboratory Standards Institute (CLSI), formerly the National Committee for Clinical Laboratory Standards (2002).

### 2.12 Minimum Inhibitory Concentration (MIC)

The minimum inhibitory concentration (MIC) of the *L. sativum* seed essential oil was determined for the microbial strains using the disc diffusion method with Muller‐Hinton Agar (MHA) and Potato Dextrose Agar (PDA), according to the method described by (Nolte 2014) with some modifications. Due to the non‐miscibility of essential oils in water, the essential oil was diluted with dimethyl sulfoxide (DMSO) to obtain concentration ranges of 30, 50, 100, and 120 μg/mL. The antimicrobial test was then carried out following the procedure outlined above.

### 2.13 In Vitro Determination of Antioxidant Activity

#### 2.13.1 Determination of Free Radical Scavenging Ability

The antioxidative property of *L. sativum* seed essential oil was assessed using the DPPH test to determine its free radical scavenging ability. The DPPH solution was prepared by solubilizing 0.05 g of DPPH in 30 mL of methanol. 100 μL of the essential oil at different concentrations was added to 2 mL of DPPH solution. The mixture was shaken vigorously for 1 min and left to stand for 30 min in the dark at room temperature. Absorbance was measured at 517 nm against a blank (DPPH/methanol). Vitamin C (ascorbic acid) was used for comparison as a standard antioxidant (Babu et al. 2011). Inhibition of free radical DPPH as a percentage [*I* (%)] was calculated as follows.

$$I\;\left(\%\right)=\frac{A_{0}-A_{1}}{A_{0}}\times 100$$

where

- *A*_0_ is the absorbance of the DPPH solution without the essential oil (the control).
- *A* is the absorbance of the DPPH solution with the essential oil.

The resulting data was presented in a graph of percent inhibition rate against sample concentration. From the plotted graph, IC_50_ was calculated.

#### 2.13.2 Phellandrene Test

The presence of phellandrene in *L. sativum* seed essential oil was tested by adding 1 mL of the essential oil into a test tube containing 2 mL of glacial acetic acid. The mixture was shaken thoroughly, and then 2 mL of a saturated aqueous solution of sodium nitrate was added dropwise. The disappearance of any crystalline mass was observed, as this would indicate the absence of phellandrene, confirming its negative presence in the essential oil (ubramanian et al. 2012).

### 2.14 Statistical Analysis

The data were analyzed using SPSS software, version 21. Significant differences between means were calculated, and the values were expressed as the mean of the three replications ± Standard Deviation (SD). Analysis of variance (one‐way ANOVA) with a *p* value < 0.05 was used to determine significant differences among treatments.

## 3 Results and Discussion

### 3.1 Physicochemical Properties of Essential Oils

The volatile seed oil extracted from three different *L. sativum* seeds by hydrodistillation were light brown in color and had a peppery‐like smell, consistent with the findings of Ravindran et al. (2012). The oil yields and physicochemical properties of *L. sativum* species obtained from dry seeds are summarized in Table 2. The yield of essential oils from the dried aerial parts of three *L. sativum* cultivars ranged from 2.31% to 3.82% (v/w) (Table 2). The yield of the *L. sativum* sample in our study was higher compared to other species within the *Lepidium* genus. For instance, Gashaw et al. (Nigussie et al. 2021) observed a yield of 2% for *L. sativum* . To the best of my knowledge, there is limited research on the chemical composition of *L. sativum* essential oil, with some reports on biological activities of various extracts, but no studies specifically addressing essential oils.

**TABLE 2:** Physicochemical characteristics of the *L. sativum* seed essential oils.

S. No.	Area	Oil yield	Specific gravity	Refractive index	Color	Phellandrene test
1	Legambo	2.31 ± 0.02	0.89 ± 0.03	1.46 ± 0.01	Brown	Negative
2	Jama	2.90 ± 0.01	0.91 ± 0.02	1.47 ± 0.02	Brown	Negative
3	Tenta	3.82 ± 0.01	0.90 ± 0.03	1.47 ± 0.01	Brown	Negative
4	Standard	—	0.78–0.97	1.42–1.51	Colorless to dark	Negative
5	LSD*α* = 0.05	0.27	0.05	0.02	—	—

*Note:* Values are mean ± standard deviation analyzed individually in triplicate.

This study reveals a significant relationship between total essential oil yields and benzyl nitrile concentration, which aligns with findings by Lucia et al. (2008). Statistical analysis of the results indicated significant differences in essential oil content across different years of the study, with the highest oil content observed in *L. sativum* collected from Tenta Woreda. This variation can be attributed to several factors that influence both the quantity and quality of essential oils, such as soil, season, temperature, geographic origin, and the ecological role of the plant organs responsible for oil production (Demuner et al. 2011).

The essential oils were found to be less dense, floating on the surface of water during hydrodistillation. The specific gravity of the oils ranged from 0.89 to 0.91, and the refractive index ranged from 1.46 to 1.47, which was within the accepted limits. Some studies have indicated that phellandrene, which can be present in some essential oils, has weak mutagenic and carcinogenic properties (Fournier et al. 1990). However, the results of our study showed that the *L. sativum* essential oil was free from phellandrene (as indicated by a negative phellandrene test), suggesting that the oil is suitable for medicinal use.

### 3.2 Chemical Composition of the Essential Oils

**FIGURE 2:**
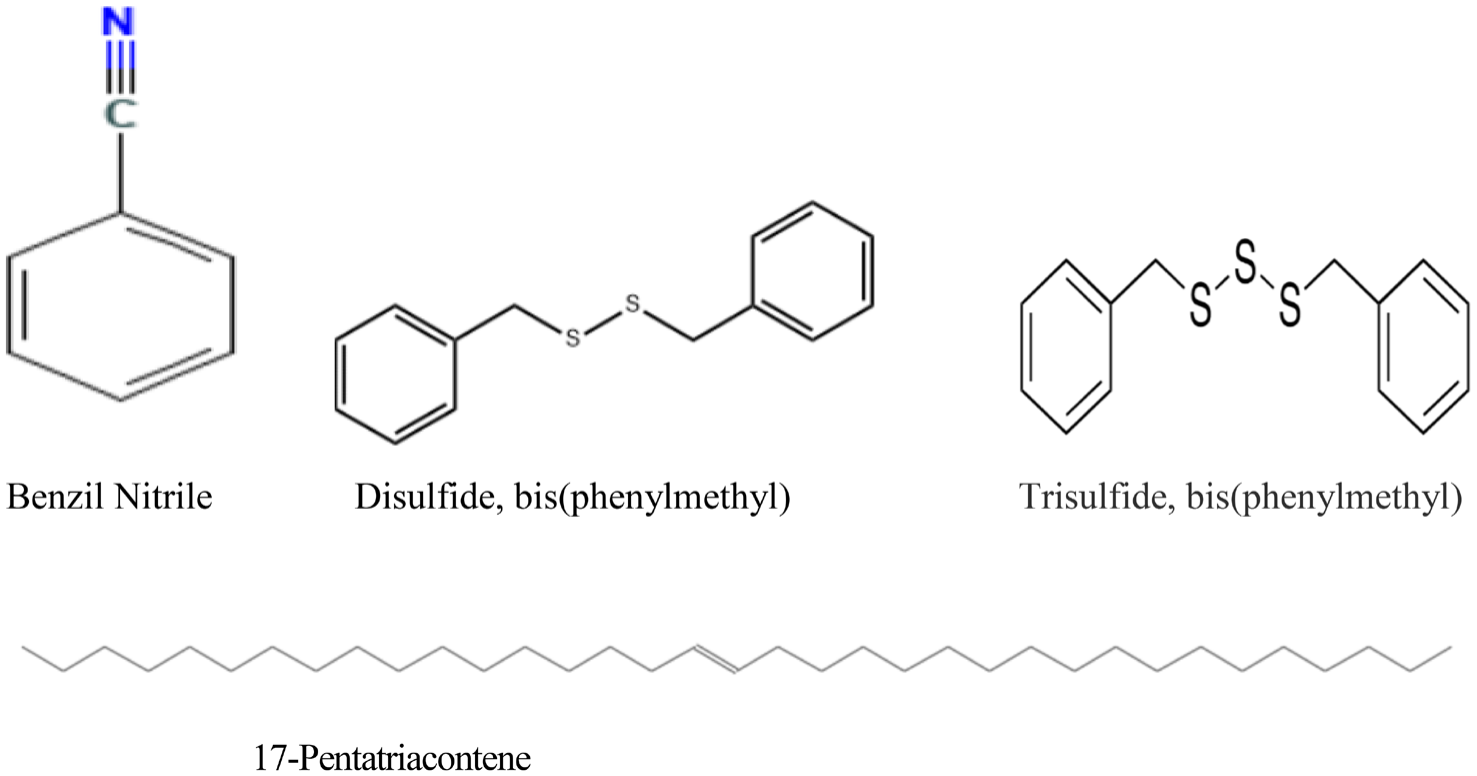
Chemical structures of the major constituents of *Lepidium sativum* seed essential oils.

The percentage composition of the obtained essential oils and the major classes of identified constituents are presented in Tables 3, 4, 5. Volatile components are listed in the order of their elution from an HP‐5 MS column to ensure consistency in compound identification and comparison. The structural representations of the principal constituents identified in *Lepidium sativum* seed essential oil are illustrated in Figure 2, providing insight into their chemical nature and potential biological activities.

**TABLE 3:** Volatile components of the essential oil of *L. sativum* seed obtained from Legambo.

S/N	Compound name	Formuls	RT (min)	Area	% Area
1	Ethylbenzene	C_8_H_10_	5.30	6,562,869	0.26
2	Styrene	C_8_H_8_	6.05	7,496,078	0.30
3	Benzene, propyl‐	C_9_H_12_	7.66	6,632,793	0.27
4	Benzaldehyde	C_7_H_6_O	8.00	25,182,677	1.01
5	Benzene, n‐butyl‐	C_10_H_14_	10.6	12,444,234	0.50
6	Benzene methane thiol	C_7_H_8_S	11.43	36,655,091	1.47
7	1‐Methyl‐2‐phenyl cyclopropane	C_10_H_12_	12.67	17,100,374	0.69
8	Benzyl nitrile	C_8_H_7_N	13.27	1.106E+09	44.43
9	Benzene, pentyl	C_11_H_16_	13.57	29,513,159	1.19
10	Benzene propanoic acid.1 ± ‐(hydroxyimino)‐	C_9_H_9_NO_3_	13.89	4,614,203	0.19
11	3‐Methyl‐4‐isopropylphenol	C_10_H_14_O	17.56	6,784,630	0.27
12	*N*‐Benzyl pyrazole	C_10_H_10_N_2_	19.03	9,555,078	0.38
13	Benzyl methyl disulfide	C_8_H_10_S_2_	19.94	2,558,806	1.03
14	Tetradecane	C_14_H_30_	20.20	4,523,686	0.18
15	Pentadecane	C_15_H_32_	22.71	7,570,452	0.30
16	2‐Phenylacetic acid	C_8_H_8_O_2_	25.56	6,978,764	0.28
17	8‐Heptadecene	C_17_H_34_	25.95	11,485,710	0.46
18	Heptadecane	C_17_H_36_	26.20	8,967,487	0.36
19	Trisulfide, bis(phenylmethyl)	C_14_H_14_S_3_	26.57	406,285,310	16.33
20	Hexadecane nitrile	C_16_H_31_N	27.89	70,948,003	2.85
21	Hexadecanoic acid, methyl ester	C_17_H_34_O_2_	28.04	85,926,768	3.45
22	17‐Pentatriacontene	C_35_H_70_	28.88	279,343,298	11.23
23	Disulfide, bis(phenylmethyl)	C_14_H_14_S_2_	29.05	312,588,182	12.56

For *L. sativum* seed collected from Legambo district, 23 compounds were identified, accounting for 99.99% of the total essential oil (Table 3). The main constituents were Benzyl nitrile (44.43%), Trisulfide, bis(phenylmethyl) (16.33%), Disulfide, bis(phenylmethyl) (12.56%), and 17‐Pentatriacontene (11.23%). Other minor components included Hexadecanoic acid, methyl ester (3.45%), Hexadecane nitrile (2.85%), Benzene methane thiol (1.47%), Benzene, pentyl (1.19%), Benzyl methyl disulfide (1.03%), and Benzaldehyde (1.01%). The rest of the components were trace amounts.

In the essential oil of *L. sativum* from Jama (Table 4), 44 compounds were identified, accounting for 99.99% of the total essential oil. The main compound was Benzyl nitrile (55.29%), with minor constituents such as Benzyl methyl disulfide (4.08%), Pyridine, 4‐(2‐phenylethyl)‐ (3.98%), Tetratriacontane (3.97%), Disulfide, bis(phenylmethyl) (3.65%), 1‐Methyl‐2‐phenylcyclopropane (2.83%), Benzene, pentyl‐ (2.15%), Benzene, *n*‐butyl‐ (1.90%), *N*‐Benzyl pyrazole (1.70%), Benzaldehyde (1.54%), Pentadecane (1.22%), Hexadecanoic acid, methyl ester (1.02%), and Benzene methane thiol (1.00%). The remainder were trace components.

**TABLE 4:** Volatile components of the essential oil of *L. sativum* seed obtained from Jama.

S/N	Compound name	Formula	RT (min)	Area	% Area
1	Ethyl benzene	C_8_H_10_	5.29	24,491,659	0.81
2	Styrene	С_8_Н_8_	6.05	27,153,030	0.90
3	1H‐Pyrrole, 1‐butyl‐	C_8_H_13_N	7.44	6,858,268	0.23
4	Веnzene, ргоруl‐	C_9_H_12_	7.65	22,006,514	0.73
5	Thiphene, 2‐ргоруl‐	C_7_H_10_S	7.71	8,309,835	0.28
6	Benzal dehyde	С_7_Н_6_О	7.97	46,335,966	1.54
7	Benzene, 1‐ethyl‐2‐methyl‐	С_9_Н_12_	8.34	9,178,290	0.31
8	Веnzеnе, сусloргоруl‐	C_9_H_10_	9.78	10,206,104	0.34
9	1H‐Pyrrole, 1‐pentyl‐	C_9_H_15_N	10.48	11,008,334	0.37
10	Benzenе, n‐butyl‐	С_10_H_14_	10.60	57,111,492	1.90
11	lndan,1‐methyl‐	С_10_Н_12_	11.26	9,359,411	0.31
12	Benzene methane thiol	C_7_H_8_S	11.43	30,195,739	1.00
13	Undecane	C_11_H_24_	11.87	11,230,587	0.37
14	5‐Undecene	С_11_H_22_	12.02	7,584,844	0.25
15	1‐Metyl‐2‐phenylcyclopгоpanе	С_10_Н_12_	12.65	85,277,671	2.83
16	Benzyl nitrile	C_8_H_7_N	13.33	1.663Е + 09	55.29
17	Benzene, pentyl‐	C_11_H_16_	13.58	64,568,135	2.15
18	Benzene propanoic acid, 1 ± (hydroxyimino)‐	C_9_H_9_NO_3_	13.92	22,898,298	0.76
19	1H‐Pyrrole, 1‐(2‐furanylmethyl)‐	C_9_H_9_NO	14.27	13,202,257	0.44
20	Bеnzene, 1‐pentenyl‐	C_11_H_14_	15.51	8,455,989	0.28
21	2‐Ethyl‐2,3‐dihydro‐1H‐indene	C_11_H_14_	15.71	8,625,305	0.29
22	Веnzene, hеху1‐	C_12_H_18_	16.48	14,576,049	0.48
23	Тridecane	С_1З_Н_28_	17.56	9,715,861	0.32
24	*N*‐Benzyl pyrazole	C_10_H_10_N_2_	19.02	51,241,149	1.70
25	Веnzene, hерtуl‐	С_13_H_20_	19.28	16,740,135	0.56
26	Benzуl methyl disulfide	C_8_H_10_S_2_	19.94	122,713,635	4.08
27	Tetradecane	C_14_H_30_	20.20	18,724,611	0.62
28	1H‐Pyrrole, 2,5‐dimethyl‐1‐phenyl‐	C_12_H_13_N	21.26	25,240,510	0.84
29	Веnzene, octyl‐	С_14_Н_22_	21.91	13,292,790	0.44
30	Cyclopentadecane	C_15_H_30_	22.37	10,226,108	0.34
31	Pentadecane	C_15_H_32_	22.71	36,804,531	1.22
32	Benzene, nonyl‐	C_15_H_24_	24.40	16,495,510	0.55
33	Z‐8‐Hexadecene	C_16_H_32_	24.63	10,718,578	0.36
34	1‐Hexadecanol	C_16_H_34_O	24.77	6,908,976	0.23
35	Hexadecane	C_16_H_34_	24.90	12,752,388	0.42
36	2‐Phenylacetic acid	C_8_H_8_O_2_	25.55	29,609,717	0.98
37	8‐Heptadecene	C_17_H_34_	25.95	26,606,724	0.88
38	Heptadecane	C_17_H_36_	26.20	21,644,740	0.72
39	1‐Nonadecene	C_19_H_38_	27.74	16,285,629	0.54
40	Hepta decane nitrile	C_17_H_33_N	27.89	21,265,265	0.71
41	Hexadecanoic acid, methyl ester	C_17_H_34_O_2_	28.04	30,614,334	1.02
42	Disulfide, bis(phenylmethyl)	C_14_H_14_S	29.05	109,723,587	3.65
43	Pyridine,4‐(2‐phenylethyl)‐	C_13_H_13_N	29.59	119,778,133	3.98
44	Tetratriacontane	C_34_H_70_	29.79	119,352,829	3.97

For *L. sativum* from Tenta (Table 5), 29 compounds were identified, representing 99.99% of the oil. The major constituent was Benzyl nitrile (74.94%), with minor components such as Benzene, pentyl‐ (3.41%), (2‐Methylcyclopropyl) benzene (2.24%), Benzyl methyl disulfide (2.09%), Benzene, (isothiocyanatomethyl)‐ (1.90%), Benzaldehyde (1.86%), Benzene, *n*‐butyl‐ (1.83%), Pentadecane (1.16%), and 8‐Heptadecene (1.11%). The remaining components were trace amounts. These data illustrate the chemical diversity of the essential oils from different *L. sativum* cultivars, with Benzyl nitrile being the dominant component across all samples.

**TABLE 5:** Volatile components of the essential oil of *L. sativum* seed obtained from Tenta.

S/N	Compound name	Formula	RT (min)	Area	% Area
1	Ethylbenzene	C_8_H_10_	5.29	13,244,200	0.86
2	Styrene	C_8_H_8_	6.05	12,545,127	0.81
3	Benzene, propyl‐	C_9_H_12_	7.64	14,069,496	0.91
4	Benzal dehyde	C_7_H_6_O	8.00	2,875,013	1.86
5	Benzene, l‐ethyl‐4‐methyl‐	C_9_H_12_	8.33	4,760,694	0.31
6	1H‐Pyrrole,1‐pentyl‐	C_9_H_15_N	10.46	7,332,912	0.48
7	Benzene, n‐butyl‐	C_10_H_14_	10.59	28,173,862	1.83
8	Benzene methane thiol	C_7_H_8_S	11.44	6,120,362	0.40
9	Undecane	C_11_H_24_	11.86	5,089,030	0.33
10	(2‐Methylcyclopropyl) benzene	C_10_H_12_	12.64	34,607,586	2.24
11	Benzyl nitrile	C_8_H_7_N	13.25	1.156E+09	74.94
12	Benzene, pentyl‐	C_11_H_16_	13.55	52,589,960	3.41
13	Benzenepropanoicacid, alpha.‐(hydroxyimino)	C_9_H_9_NO_3_	13.87	8,234,555	0.53
14	Benzene, hexyl‐	C_12_H_18_	16.46	8,239,183	0.53
15	4‐Nonene,5‐butyl‐	C_13_H_26_	17.32	5,312,697	0.34
16	Tridecane	C_13_H_28_	17.54	5,539,096	0.36
17	*N*‐Benzylpyrazole	C_10_H_10_N_2_	19.02	8,074,803	0.52
18	Benzene, heptyl‐	C_13_H_20_	19.25	5,552,963	0.36
19	Benzene, (isothiocyanatomethyl)‐	C_8_H_7_NS	19.39	29,335,549	1.90
20	Benzyl methyl disulfide	C_8_H_10_S_2_	19.93	32,275,108	2.09
21	Tetradecane	C_14_H_30_	20.18	6,096,907	0.40
22	Benzene, octyl‐	C_14_H_22_	21.91	5,002,260	0.32
23	Pentadecane	C_15_H_32_	22.69	17,854,083	1.16
24	Benzene, nonyl‐	C_15_H_24_	24.38	5,776,903	0.37
25	Z‐8‐Hexadecene	C_16_H_32_	24.61	4,743,339	0.31
26	Hexadecane	C_16_H_34_	24.88	4,796,052	0.31
27	2‐Phenylacetic acid	C_8_H_8_O_2_	25.55	6,640,302	0.43
28	8‐Heptadecene	C_17_H_34_	25.93	17,062,214	1.11
29	Heneicosane	C_21_H_44_	26.19	8,849,230	0.57

In this study, Benzyl nitrile emerged as the predominant component in the essential oils extracted from garden cress seeds. This observation is consistent with the findings of Sharma et al. (2015), who identified high levels of Benzyl nitrile in *L. sativum* essential oils across various regions. The consistent presence of this compound underscores its significance as a bioactive element contributing to the therapeutic potential of *L. sativum* essential oils. The essential oils extracted from the garden cress seeds revealed notable concentrations of Trisulfide, bis(phenylmethyl) and Disulfide, bis(phenylmethyl) as major components. The variability of these sulfur‐containing compounds has been previously noted by Gholamzadeh and Khosravi (2016), who suggested that environmental factors play a crucial role in influencing their concentrations. This finding illustrates the diverse chemical profile that can exist within the same species based on geographical differences. The identification of 17‐Pentatriacontene as a major component in the essential oil represents a unique finding, as this compound is infrequently reported in the literature. Kumar et al. (2019) highlighted the presence of similar unusual hydrocarbons in *L. sativum* from specific locales, suggesting that regional characteristics can significantly impact the chemical diversity of essential oils. This adds to the complexity of understanding the full spectrum of bioactive compounds present in *L. sativum* .

In contrast to the current study, previous research conducted by Moghadam and Alizadeh (2017) reported high levels of Allyl isothiocyanate, a compound widely associated with the medicinal properties of *L. sativum* . The absence of this compound in the essential oils analyzed in this study may indicate geographical or genetic divergences that affect the overall composition. This discrepancy highlights the necessity for further studies to explore the factors that contribute to variations in essential oil profiles among different populations of *L. sativum* .

Benzyl nitrile was the major component of *L. sativum* seed essential oils from the Legambo, Jama, and Tenta cultivars. Benzyl nitrile is a versatile and widely utilized organic compound with significant applications in various industries. It is a colorless liquid with a pleasant yet sharp odor and is soluble in organic solvents. As an intermediate compound, benzyl nitrile plays a crucial role in the synthesis of a variety of organic compounds.

Its importance extends to the production of biologically active compounds, including antifungal agents, antibiotics, and antiviral agents. Beyond its pharmaceutical and biomedical applications, benzyl nitrile is also employed in the creation of diverse chemicals, such as dyes, as well as in the manufacture of polymers like polyamides and polyurethanes. This makes benzyl nitrile not only essential in scientific research but also valuable in industrial applications where it is used to produce a wide range of functional and commercial products. https://www.scbt.com/p/benzeneacetonitrile‐140‐294?srsltid=AfmBOoq0HSTaxAHQr1mmwuokMRpcXtChocDqg6v9eAAxON5B_tilaccp.

The essential oils derived from *L. sativum* seeds exhibit a broad spectrum of pharmacological activities, highlighting their potential as a source of bioactive compounds. The analysis also revealed that the seed oil contains a diverse array of chemical compounds, including saturated and unsaturated fatty acids, nitrogen‐ and sulfur‐containing aromatic derivatives, saturated hydrocarbons, and nitrogen‐ and oxygen‐containing aromatic derivatives, as well as nitrogen‐containing ketones.

Statistical analysis (Table 6) showed that the essential oils from all three cultivation areas were primarily characterized by a high content of benzyl nitrile, ranging from 44.43% to 74.94%. Thirteen compounds were common across the essential oils from the different regions, although their concentrations varied. This variation is statistically significant, indicating differences among the treatments. The differences in chemical composition can be attributed to various factors, such as seasonality, circadian rhythms, the age and development of the plant, temperature, water availability, ultraviolet radiation, nutrient content, altitude, atmospheric pollution, and pathogen attacks. These factors can significantly influence both the yield and composition of essential oils, as reflected in the differences observed between studies and different species (Gobbo‐Neto and Lopes 2007; Dudareva et al. 2004).

**TABLE 6:** Statistical analysis of the common constituents of *L. sativum* seed essential oil in the respective regions.

No.	Compounds	RT (min)	Legambo	Jama	Tenta	LSD*α* = 0.05
1	Ethyl benzene	5.30	0.26 ± 0.02	0.81 ± 0.01	0.86 ± 0.02	0.03
2	Styrene	6.05	0.30 ± 0.01	0.90 ± 0.01	0.81 ± 0.01	0.02
3	Benzaldehyde	8.00	1.01 ± 0.06	1.54 ± 0.04	1.86 ± 0.04	0.09
4	Benzene methane thiol	11.43	1.47 ± 0.03	1.00 ± 0.02	0.4 ± 0.1	0.12
5	1‐Methyl‐2‐phenyl cyclopropane	12.67	0.69 ± 0.01	2.83 ± 0.02	2.24 ± 0.01	0.28
6	Benzyl nitrile	13.27	44.43 ± 0.02	55.29 ± 0.01	74.94 ± 0.62	0.69
7	Benzene, pentyl‐	13.57	1.19 ± 0.06	2.15 ± 0.03	3.41 ± 0.02	0.09
8	Benzenepropanoic acid, alpha.‐(hydroxyimino)	13.89	0.19 ± 0.01	0.76 ± 0.01	0.53 ± 0.01	0.02
9	*N*‐Benzyl pyrazole	19.03	0.38 ± 0.02	1.70 ± 0.01	0.52 ± 0.03	0.20
10	Benzyl methyl disulfide	19.94	1.03 ± 0.01	4.08 ± 0.02	2.09 ± 0.03	0.13
11	Tetradecane	20.20	0.18 ± 0.03	0.62 ± 0.02	0.40 ± 0.15	0.07
12	2‐Phenyl acetic acid	25.56	0.28 ± 0.02	0.98 ± 0.06	0.43 ± 0.02	0.11
13	8‐Heptadecene	25.95	0.46 ± 0.01	0.88 ± 0.03	1.11 ± 0.01	0.04

*Note:* ±Standard deviation (*n* = 3).

### 3.3 Biological Activities of the Essential Oils

#### 3.3.1 Antimicrobial Activity

The antimicrobial activity of *Lepidium sativum* seed essential oil was evaluated by determining its bactericidal and fungicidal effects, as well as the minimum inhibitory concentration (MIC) against a panel of selected bacterial and fungal strains, using the filter paper disc and disc diffusion methods following standardized protocols to ensure reproducibility. The potency of the essential oil was assessed based on the size of the inhibition zones and MIC values, and the comprehensive results are presented in Tables 7 and 8, which summarizes the antimicrobial activity of *Lepidium sativum* seed essential oil against the selected strains, expressed as inhibition zone diameters (mm) and MIC values (μg/mL).

**TABLE 7:** Zone of inhibition (mm) of antimicrobial activities of *L. sativum* seed essential oils.

Microorganisms	Average zone of inhibition of essential oils collected from			Positive controls		LSD*α* = 0.05
	Legambo	Jama	Tenta	Gentamicin	Clotrimazole	
*S. aureus*	29.0 ± 0.4	32.3 ± 0.5	36.8 ± 0.5	27.4 ± 0.9	—	1.01
*B. subtilis*	30.7 ± 0.1	32.1 ± 0.4	34.8 ± 0.6	27.0 ± 0.2	—	1.05
*E. coli*	27.2 ± 0.4	29.3 ± 0.4	33.3 ± 0.6	24.7 ± 0.1	—	0.93
*P. multocida*	28.5 ± 0.4	30.6 ± 0.2	33.9 ± 0.2	25.8 ± 0.1	—	0.61
*C. albicans*	33.5 ± 0.2	31.2 ± 0.7	29.7 ± 0.5	—	28.3 ± 0.7	0.56

**TABLE 8:** Minimum inhibitory concentration (MIC) mg/L.

Microorganisms	Average zone of inhibition of essential oils collected from			Positive controls		LSD*α* = 0.05
	Legambo	Jama	Tenta	Gentamicin	Clotrimazole	
*S. aureus*	1.26 ± 0.02	2.23 ± 0.01	2.41 ± 0.01	3.6 ± 0.01	—	0.03
*B. subtilis*	1.64 ± 0.05	2.39 ± 0.02	2.58 ± 0.01	3.9 ± 0.06	—	0.14
*E. coli*	2.51 ± 0.01	2.72 ± 0.01	2.91 ± 0.02	4.4 ± 0.01	—	0.02
*P. multocida*	2.55 ± 0.01	2.81 ± 0.02	3.01 ± 0.02	4.2 ± 0.05	—	0.09
*C. albicans*	2.01 ± 0.05	2.14 ± 0.01	2.62 ± 0.06	—	3.52 ± 0.03	0.06

The findings demonstrated that the pure *L. sativum* seed essential oil exhibited significant antimicrobial activity against all the tested bacteria and fungi. The essential oil showed strong activity across different regions, with larger inhibition zones (27.2–36.8 mm for bacteria and 29.7–33.5 mm for fungi) and smaller MIC values (1.26–3.00 mg/L for bacteria and 2.01–2.62 mg/L for fungi) against various microbial strains. The results revealed that *L. sativum* oil had the highest antibacterial activity against *Staphylococcus aureus* (Gram‐positive), with the largest inhibition zone (36.8 mm) and the smallest MIC value (2.41 mg/L). This antibacterial effect was found to be superior to that of the antibiotic gentamicin, which showed an inhibition zone of 27.4 mm and an MIC of 3.6 mg/L.

Regarding antifungal activity, *Candida albicans* was the most sensitive microorganism, exhibiting the largest inhibition zone (33.3 mm) and the lowest MIC value (2.01 mg/L). This antifungal activity was comparable to that of the antifungal agent clotrimazole, which had an inhibition zone of 28.3 mm and an MIC of 3.5 mg/L.

In comparison to previous studies, Fikremariam et al. (Adera et al. 2022) tested petroleum ether extracts of *L. sativum* seeds and reported a maximum inhibition zone of 18.5 mm against *S. aureus* and a smaller inhibition zone of 12.57 mm against *C. albicans* . Similarly, Berehe and Boru (2014) reported inhibitory activity of *L. sativum* seed chloroform/methanol crude extracts, with a maximum inhibition zone of 17.43 mm against *S. aureus* and a minimum zone of 12.13 mm against *Escherichia coli* .

Overall, the results from this study highlight the potent antimicrobial properties of *L. sativum* seed essential oil, suggesting its potential as an effective natural antimicrobial agent.

The study conducted by Adam et al. (2011) investigated the antimicrobial activity of various extracts of *L. sativum* seed from Sudan, including petroleum ether, aqueous, and methanolic extracts, against six opportunistic microorganisms: *S. aureus* , *E. coli* , *K. pneumoniae* , *Proteus vulgaris* , *P. aeruginosa* , and the fungus *C. albicans* . The findings indicated that petroleum ether, at concentrations of 2.5%, 5%, and 10%, was a more effective solvent for extracting antimicrobial substances from *L. sativum* seeds than methanol and water.

In contrast, other studies, such as the one conducted in Ethiopia (Berehe and Boru 2014), showed that crude extracts from *L. sativum* seeds exhibited antimicrobial activity against fungi ( *A. niger* , *F. oxysporum*, and *F. solani*) and bacteria ( *E. coli* , *S. typhi* , *B. subtilis* , and *S. aureus* ). However, one notable limitation in those studies was the lack of Minimum Inhibitory Concentration (MIC) data, which makes it difficult to compare the antimicrobial potency of the extracts with the results from this current study, where MIC values were determined.

The antimicrobial activity of *L. sativum* seed essential oil observed in this study demonstrated stronger activity against Gram‐positive bacteria, with mean inhibition zones of 29–36.8 mm and MIC values ranging from 1.26 to 2.58 mg/L, compared to Gram‐negative bacteria, which had inhibition zones ranging from 27.2 to 33.9 mm and MIC values between 2.51 and 3.00 mg/L. This is consistent with the findings from other studies (Abo El‐Maati et al. 2016; Hussain et al. 2010; Lodhia et al. 2009), where Gram‐positive bacteria were generally more susceptible to essential oils than Gram‐negative bacteria. The higher resistance of Gram‐negative bacteria to essential oils is often attributed to the outer membrane surrounding the cell wall, which acts as a barrier, limiting the diffusion of hydrophobic compounds, such as those found in essential oils, through the lipopolysaccharide layer (Burt 2004).

Furthermore, studies investigating essential oils' antimicrobial effects on food spoilage organisms and foodborne pathogens have also commonly found that essential oils tend to be more effective against Gram‐positive bacteria. However, not all studies have concluded this, as Gram‐negative bacteria, such as *A. hydrophila* , have been found to be highly susceptible to essential oils in some cases (Canillac and Mourey 2001; Cimanga et al. 2002; Wilkinson et al. 2003; Stecchini et al. 1993). Additionally, certain studies have noted that while no significant difference was found between Gram‐positive and Gram‐negative bacteria after 24 h, the inhibitory effect of essential oils tends to last longer against Gram‐negative bacteria, with the effect often extending to 48 h (Quattara et al. 1997).

These results underline the complex nature of essential oils' antimicrobial activities and highlight the importance of considering both bacterial type and oil composition when evaluating their potential uses in antimicrobial applications.

The findings of this study suggest that the essential oil extracted from *L. sativum* seeds possesses significant antimicrobial activity, primarily attributed to the presence of benzyl nitrile, which was identified as the major active compound against both Gram‐positive and Gram‐negative bacteria, as well as dermatophytic fungi. As noted by Scotti and Barlow (2022), benzyl nitrile is a key antimicrobial agent in *L. sativum* seed essential oil. However, the full antimicrobial potential of the oil cannot be explained solely by the presence of its major components. Minor compounds in the oil also appear to play an essential role in its overall effectiveness (Paster et al. 1995).

Sulfur‐containing compounds, such as benzene, methane thiol, trisulfide, bis(phenylmethyl), and disulfide, bis(phenylmethyl), were found to be significant contributors to the antimicrobial activity of the essential oil. These compounds, which contain sulfur–sulfur bonds, have been shown in previous studies, such as that by Blume et al. (2023), to exhibit strong antibacterial properties, further supporting the idea that sulfur‐containing molecules contribute significantly to the antimicrobial effects observed in this study. The complex mixture of phytochemicals found in the oil implies that the antimicrobial activity could result from both the individual actions of the active compounds and possible synergistic effects between the minor components (Cowan 1999; Elyemni et al. 2020).

The antimicrobial results presented in Tables 7 and 8 demonstrate that *L. sativum* seed essential oil displayed robust activity against a range of bacterial and fungal pathogens, particularly Gram‐positive and Gram‐negative bacteria, as well as dermatophytic fungi. This aligns with the plant's traditional use in folk medicine and suggests that it could serve as a valuable alternative therapeutic agent for treating microbial infections, particularly those affecting the skin.

The GC/MS analysis revealed that between 44.43% and 74.94% of the essential oil composition was identified as benzyl nitrile, reinforcing the compound's role as a key contributor to the antimicrobial effects of the oil. The combination of benzyl nitrile and the minor components likely works synergistically to enhance the overall antimicrobial potency, making *L. sativum* seed essential oil an effective and promising candidate for further exploration in both medicinal and industrial applications.

The statistically significant differences in the antimicrobial efficacy of *L. sativum* seed essential oils from the Legambo, Jama, and Tenta districts (*p* < 0.05), as presented in Tables 7 and 8, suggests that the variations in antimicrobial activity can be attributed to the differences in the chemical composition of the oils. This is consistent with previous findings by Saliu (2011), who emphasized that both qualitative and quantitative variations in the chemical components of essential oils can significantly influence their biological activities.

The differences in antimicrobial activity observed between the oils from the different districts may be due to several factors, including the distinct chemical profiles of each oil. These profiles are influenced by factors such as geographical location, soil composition, climate, and other environmental variables. Additionally, the proportions of key components, including benzyl nitrile and other minor compounds, as well as potential synergistic interactions between the components, likely play an important role in the observed differences in antimicrobial efficacy.

Thus, it can be concluded that the geographical origin and environmental conditions in which *L. sativum* is grown could impact the overall chemical composition and biological activity of its essential oil. This underscores the importance of considering these factors when evaluating the potential applications of plant‐derived essential oils in medicine and other industries. Further studies, including detailed chemical analysis and testing across different regions, would be valuable in optimizing the use of *L. sativum* seed essential oil as a natural antimicrobial agent.

#### 3.3.2 Antimicrobial Action of Major Compounds

The major compounds present in *L. sativum* , particularly Benzyl nitrile, Trisulfide, bis(phenylmethyl), Disulfide, bis(phenylmethyl), and 17‐Pentatriacontene, exhibit significant antimicrobial activity through various mechanisms that enhance their therapeutic potential. Benzyl nitrile has been shown to disrupt bacterial cell membranes, leading to cell lysis, and it inhibits vital metabolic processes necessary for bacterial growth and reproduction (Sharma et al. 2015). This ability to compromise cell integrity makes Benzyl nitrile a promising candidate for natural antimicrobial agents.

Trisulfide, bis(phenylmethyl), is another compound that demonstrates antimicrobial effects by modifying thiol groups in bacterial proteins, which impairs their functionality and interferes with essential enzymatic activities. This disruption is crucial for the survival and proliferation of bacteria, reinforcing the compound's role as an effective antimicrobial agent (Gholamzadeh and Khosravi 2016). Similarly, disulfide, bis(phenylmethyl) acts through analogous mechanisms, targeting thiol groups and thereby affecting bacterial cellular functions, which further enhances its antimicrobial efficacy.

The identification of 17‐Pentatriacontene adds another layer of complexity to the antimicrobial profile of *L. sativum* . This hydrocarbon can integrate into the lipid membranes of microbial cells, altering their fluidity and permeability, which inhibits microbial growth by disrupting essential cellular processes (Kumar et al. 2019). This unique mechanism of action highlights the diverse strategies that *L. sativum* compounds employ to combat microbial pathogens effectively. Together, these compounds from *L. sativum* demonstrate a multifaceted approach to inhibiting microbial pathogens, showcasing their potential as natural alternatives to synthetic antimicrobial agents. The diverse mechanisms of action underscore the importance of further research into these bioactive compounds for potential application in pharmaceutical and food preservation industries.

### 3.4 Antioxidant Activities of Essential Oils

The evaluation of *Lepidium sativum* seed essential oils for their antioxidant potential using the DPPH assay revealed a dose‐dependent increase in free radical scavenging activity with increasing concentrations of the essential oil. The DPPH assay is based on the ability of antioxidants to donate electrons or hydrogen atoms to the DPPH• (purple color) radicals, thereby reducing them to DPPH‐H (yellow color). The extent of this reduction is observed as a decrease in absorbance at 517 nm, allowing for the measurement of the antioxidant activity.

The IC_50_ value, which is the concentration required to inhibit 50% of DPPH radical scavenging, serves as a measure of antioxidant potency. A lower IC_50_ indicates stronger antioxidant activity. The results from this study suggest that the essential oil of *L. sativum* exhibited good scavenging activity, with the percentage inhibition of DPPH radicals increasing as the concentration of the essential oil was raised. While the antioxidant activity was notable, the essential oil's scavenging effect was somewhat weaker compared to ascorbic acid, which is commonly used as a positive control for antioxidant activity.

In general, an inhibition percentage of 71%–100% is considered strong, 41%–70% moderate, and ≤ 40% weak, based on previous research (Devi 2009). The essential oil demonstrated a strong scavenging activity when tested at concentrations as high as 5 mg/mL, which was consistent with its effectiveness as an antioxidant. The findings further support the potential of *L. sativum* seed essential oil as a natural antioxidant, although its activity is not as potent as that of ascorbic acid. This study contributes to the growing body of evidence suggesting that *L. sativum* essential oil could have applications in preserving food or as a component in products targeting oxidative stress‐related conditions.

**FIGURE 3:**
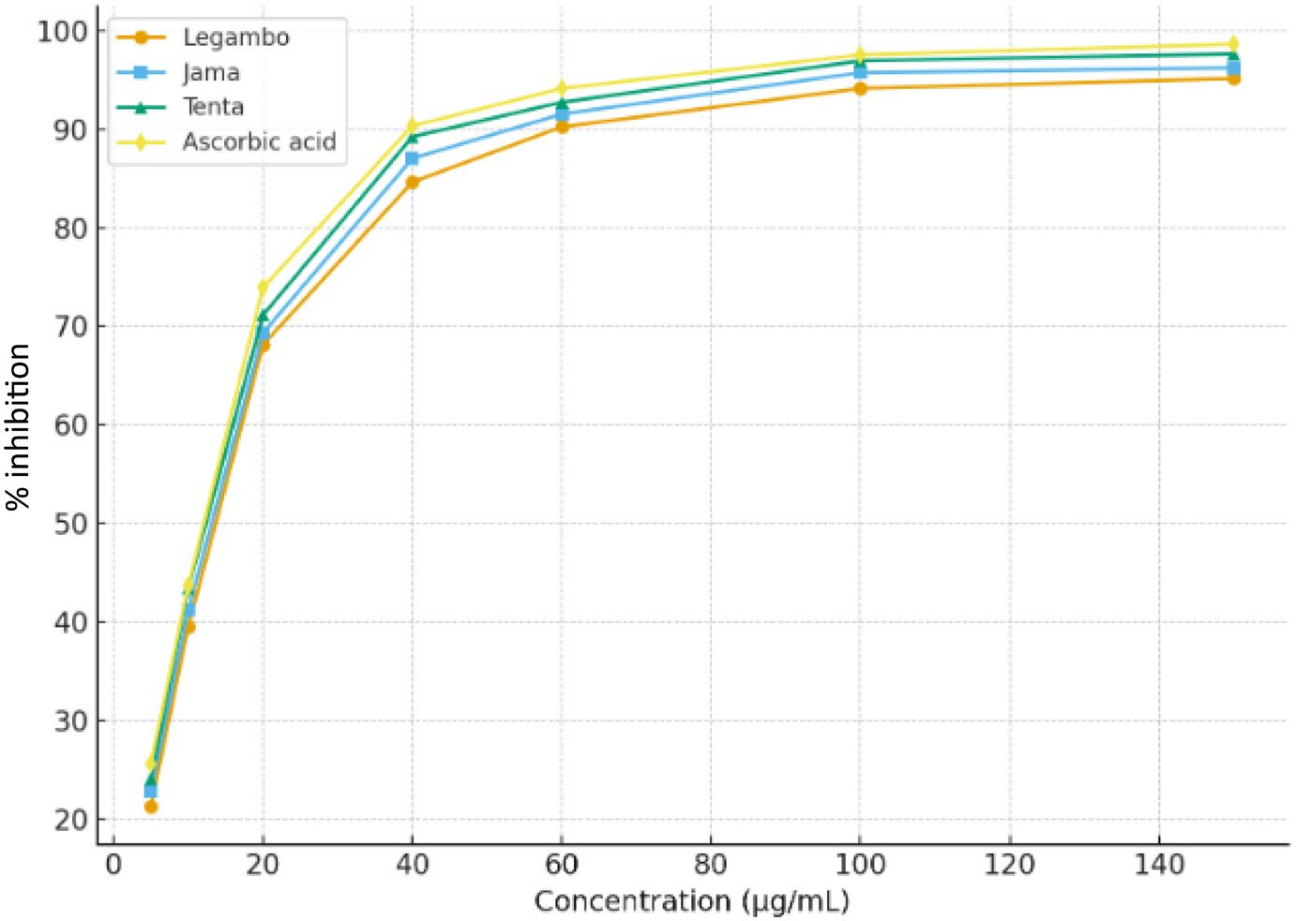
Free radical scavenging activity of *L. sativum* seed essential oils growing in different regions and ascorbic acid.

The data from the DPPH assay, presented in Tables 9 and 10 and illustrated in Figure 3, demonstrate that the antioxidant potential of *Lepidium sativum* seed essential oils increases with concentration. At the highest concentration tested (150 μg/mL), the oils exhibited pronounced inhibition of DPPH radicals, with values ranging from 95.1% to 96.7% depending on the cultivation region. In contrast, the lowest concentration (5 μg/mL) resulted in substantially lower inhibition, ranging from 21.3% to 24.1%, thereby confirming a clear dose‐dependent antioxidant activity.

**TABLE 9:** The percentage inhibition of various concentrations of *L. sativum* seed essential oils and ascorbic acid.

Concentration (μg/mL)	% Inhibition of essential oils collected from			% Inhibition of ascorbic acid	LSD*α* = 0.05
	Legambo	Jama	Tenta		
5	21.3 ± 0.4	22.8 ± 0.2	24.1 ± 0.5	25.6 ± 0.6	0.24
10	39.5 ± 1.2	41.2 ± 0.7	43.4 ± 0.9	43.7 ± 0.1	0.37
20	68.1 ± 2.1	69.3 ± 0.6	71.1 ± 0.8	73.9 ± 0.9	0.29
40	84.6 ± 0.7	87.0 ± 0.3	89.2 ± 0.2	90.3 ± 0.3	0.53
60	90.2 ± 0.2	91.5 ± 0.1	92.7 ± 0.3	94.1 ± 0.2	0.41
100	94.1 ± 0.2	95.7 ± 0.1	96.9 ± 0.8	97.5 ± 0.1	0.38
150	95.1 ± 0.2	96.2 ± 0.2	97.6 ± 0.4	98.6 ± 0.7	0.57

**TABLE 10:** DPPH test (IC_50_ mg/mL) of *L. sativum* seed essential oils growing in different regions.

Parameters	Legambo	Jamma	Tenta	Ascorbic acid	LSD*α* = 0.05
DPPH (IC_50_ μg/mL)	12.1 ± 0.8	11.7 ± 0.1	11.3 ± 0.1	10.9 ± 0.6	0.09

The study further calculated the IC_50_ values, which ranged from 11.3 to 12.1 μg/mL, and these values were used to compare the antioxidant potency of the oils. A lower IC_50_ value indicates a stronger antioxidant effect, and as such, the essential oil obtained from Tenta exhibited the strongest antioxidant activity (IC_50_ = 11.3 ± 0.1 μg/mL), followed by the oil from Jama (IC_50_ = 11.7 ± 0.1 μg/mL) and the oil from Legambo (IC_50_ = 12.1 ± 0.8 μg/mL). Statistically significant differences (*p* < 0.05 based on LSD) were observed among the oils from the different regions, indicating that regional factors such as soil composition, climate, and other environmental factors may influence the antioxidant activity of the essential oils.

The findings in this study suggest that the *L. sativum* seed essential oils from the Tenta region are particularly potent antioxidants when compared to oils from Jama and Legambo. The regional variations in antioxidant activity may be attributed to differences in the antioxidant compounds present in the oils (Yong 2015), as well as regional environmental factors that could affect the plants' chemical profiles, a notion supported by Msaada et al. (2015).

Moreover, when compared to other studies, the IC_50_ values obtained in this study are considerably lower than those reported by Umesh et al. (IC_50_ = 25 mg/mL) (Umesha and Naidu 2015), Ahamad et al. (IC_50_ = 62 mg/mL) (Ahamad et al. 2015), and Chatoui et al. (IC_50_ = 925 mg/mL) (Chatoui et al. 2016), which further emphasizes the strong antioxidant potential of *L. sativum* seed essential oil in this study. This suggests that the oil from *L. sativum* seeds could be a promising natural antioxidant source, with potential applications in food preservation and health‐related products.

### 3.5 Antioxidant Action of Major Compounds

The major compounds in *L. sativum* , including Benzyl nitrile, Trisulfide, bis(phenylmethyl), Disulfide, bis(phenylmethyl), and 17‐Pentatriacontene, exhibit notable antioxidant activity through various mechanisms that contribute to their protective effects against oxidative stress. Benzyl nitrile has been identified as an effective scavenger of free radicals, neutralizing reactive oxygen species (ROS) and thus reducing oxidative damage to cellular components (Sharma et al. 2015). Similarly, Trisulfide, bis(phenylmethyl) functions as an antioxidant by acting as an electron donor, which helps to neutralize free radicals and mitigate oxidative stress in biological systems (Gholamzadeh and Khosravi 2016). Disulfide, bis(phenylmethyl) also contributes to antioxidant defense by participating in redox reactions that help maintain the balance between oxidants and antioxidants within cells. Additionally, 17‐Pentatriacontene, while primarily noted for its antimicrobial properties, may also enhance antioxidant activity by reinforcing cellular membranes and preventing lipid peroxidation, thereby protecting cells from oxidative damage (Kumar et al. 2019). Collectively, these compounds reveal a comprehensive antioxidant profile, highlighting their potential for therapeutic applications in mitigating oxidative stress‐related diseases.

## 4 Conclusions

This study on the essential oils extracted from *Lepidium sativum* seeds across three different districts—Legambo, Jama, and Tenta—provides valuable insights into their chemical composition, antimicrobial, and antioxidant properties, as well as their potential medicinal applications. The oils were primarily composed of benzyl nitrile, along with other sulfur‐containing compounds, fatty acids, and hydrocarbons, which collectively contribute to their therapeutic potential. Significant regional variations were observed in both the yield and composition of the essential oils.

The essential oils exhibited strong antimicrobial activity against both Gram‐positive and Gram‐negative bacteria, as well as fungi, with benzyl nitrile and sulfur‐containing compounds being the key contributors to this bioactivity. The oils also demonstrated impressive antioxidant properties, with oils from the Tenta district showing the strongest activity, suggesting their potential as natural antioxidants in various applications. The findings underscore the promising pharmacological potential of *L. sativum* essential oils, particularly in the areas of antimicrobial and antioxidant therapies.

## Author Contributions

**Melese Damtew Asfaw:** conceptualization (equal), data curation (equal), formal analysis (equal), funding acquisition (equal), investigation (equal), methodology (equal), software (equal), supervision (equal), validation (equal), visualization (equal), writing – original draft (equal), writing – review and editing (equal). **Mequanint Gebeyehu Awoke:** methodology (equal), visualization (equal), writing – review and editing (equal). **Adamu Tizazu Yadeta:** funding acquisition (equal), supervision (equal), visualization (equal), writing – review and editing (equal).

## Conflicts of Interest

The authors declare no conflicts of interest.

## Data Availability

The data that support the findings of this study are available on request from the corresponding author.
